# Supplementary material for: A UMLS-based spell checker for natural language processing in vaccine safety
Source: BMC Med Inform Decis Mak. 2007 Feb 12;7:3. doi: 10.1186/1472-6947-7-3 (PMC1805499; doi:10.1186/1472-6947-7-3)
Supplement: Additional file 13 — RAP application directory. Sets up directory for RAP (RDF application for PHP) source code [file 1472-6947-7-3-S13.gz › rap/doc/phpdoc/model/_model_MemModel_php.html]

xml version="1.0" encoding="iso-8859-1"?


Docs for page MemModel.php


## /model/MemModel.php

Description

Description |
Classes

- filesource: Source Code for this file

Classes

Description |
Classes

| Class | Description |
| --- | --- |
| MemModel | A MemModel is an RDF Model, which is stored in the main memory. |

Documentation generated on Thu, 7 Jul 2005 13:41:52 +0200 by phpDocumentor 1.3.0RC3
